# Natalia Tomilina – personality and profession

**DOI:** 10.1186/s12882-019-1303-4

**Published:** 2019-04-01

**Authors:** Elena Zakharova

**Affiliations:** 1 Nephrology, Moscow City Hospital n.a. S.P. Botkin, Moscow, Russian Federation; 2 Nephrology, Moscow State University of Medicine and Dentistry, Moscow, Russian Federation; 3 Nephrology, Russian Medical Academy of Continuous Professional Education, Moscow, Russian Federation

## Abstract

Natalina Tomilina, a pioneer of Russian nephrology, is a clinician, researcher, teacher, organizer, leader, and a real pioneer, who has worked in nephrology from the very beginning of its development in Russia and continues to inspire new generations of Russian nephrologists. Her interests are very broad: from the physiology and pathophysiology of water and electrolyte balance and tubular dysfunctions to the management of transplant rejection, and from nephropathology to the treatment of idiopathic glomerulonephritis and ANCA-associated vasculitis…. to name a few. She implemented peritoneal dialysis, started first ICU for kidney patients in Russia, opened the door for the international communications, initiated a registry of the patients receiving RRT, and she never stopped seeing patients with kidney problems. In the interview on can find not only the story of her professional life, but also standpoint and philosophy of a great personality. Answering the question about emigration she said: “I never wanted to leave – I have to work at home, where I know and understand almost everything about my patients. Don’t talk about prosperity, prosperous life sooner or later becomes boring. Prosperity is not the main point, and this is not prosperity, what gives you satisfaction. I feel that one should live in the place where he or she has an opportunity for personal fulfillment ad maximum”. Her personal fulfillment is 100 % indeed.

## Editorial

**Fig. 1:**
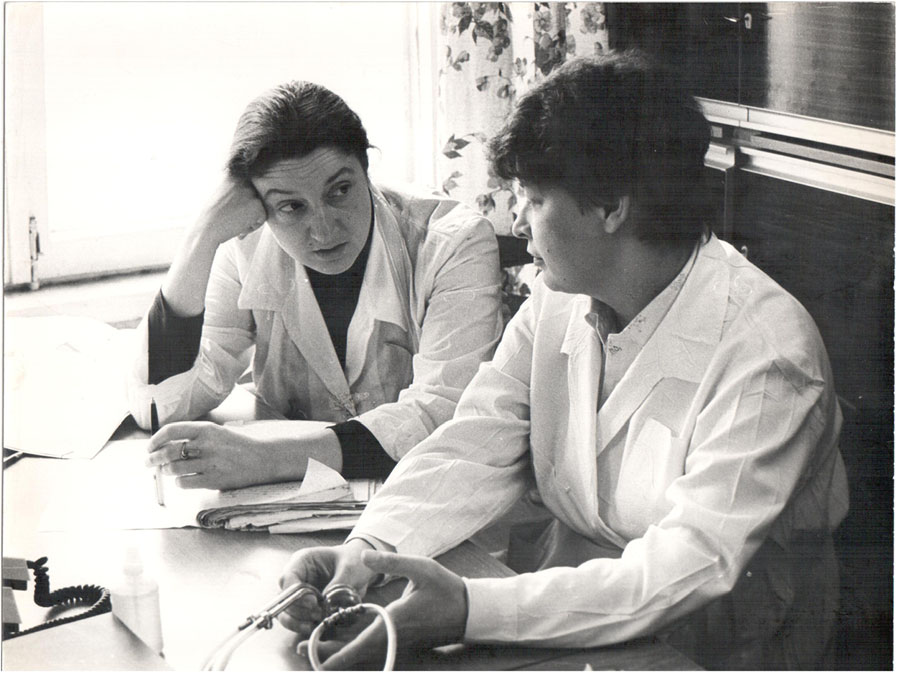
Natalia Tomilina (right) and Elena Zakarova discussing a clinical case: Mosсow 1983

I am delighted to contribute to the ‘Pioneers of Nephrology’ collection a portrait of Natalia Tomilina, one of the pioneers of Russian nephrology, describing her professional achievements and personality. She was my mentor from the time I took my first steps in nephrology, and I have learned a great deal from her (Fig. 1).

**Fig. 2:**
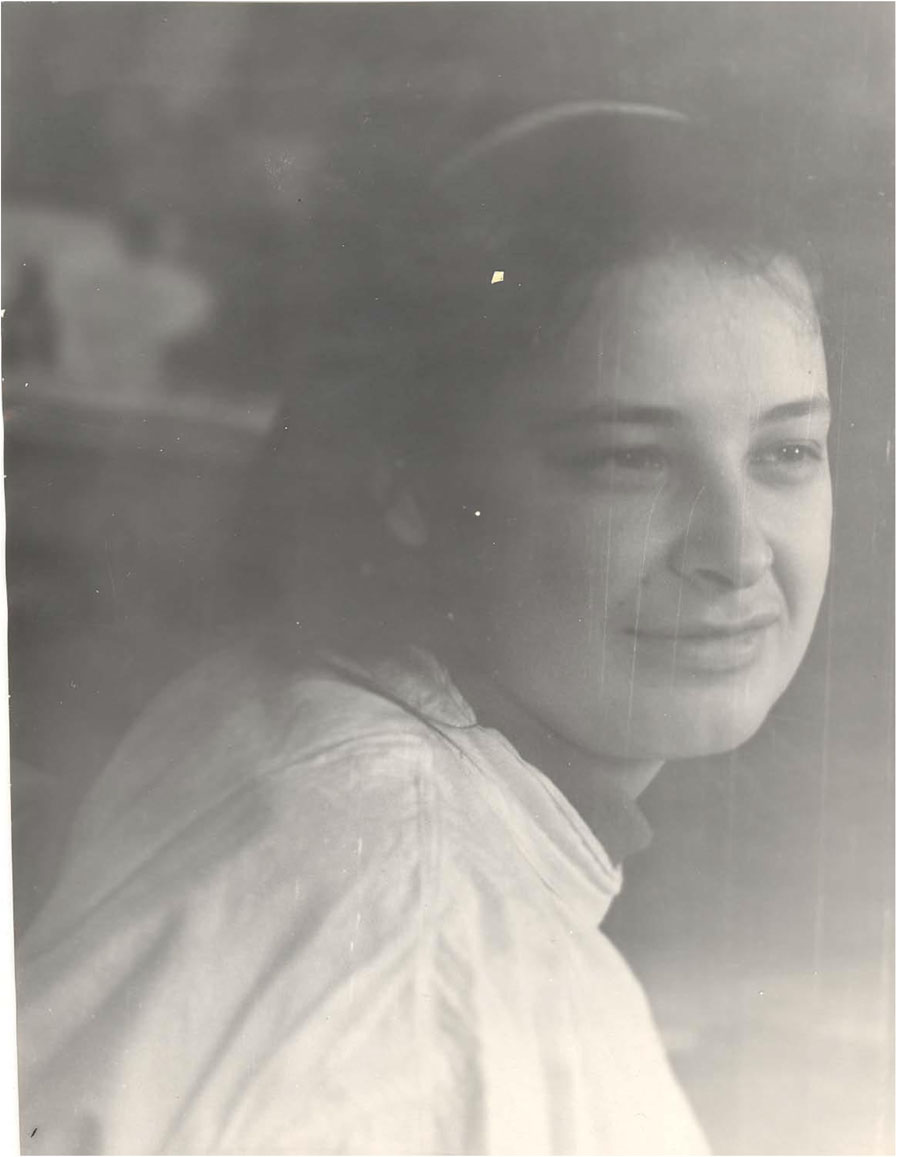
Natalia Tomilina as a young doctor (1963)

**Fig. 3:**
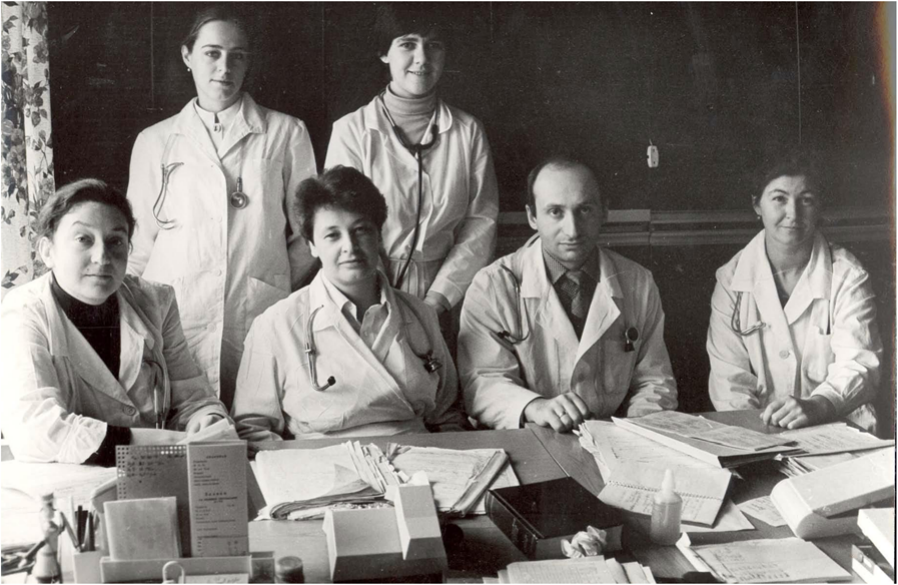
Natalia Tomilina, sitting in the middle, and her team, Moscow 1983

Natalia Tomilina was born in the city of Serpukhov, near Moscow, in a medical family. Her father was a pediatrician, but during the Second World War he was called up to serve as a doctor in the army and then, after the end of the war, he signed up to work in the army and continued his service as a military doctor. He even worked in Germany for several years. Natalia’s mother was also a physician but when the family moved to Germany, she stopped practicing medicine. Natalia’s second language was German, and only when she became a nephrologist, did she study English, first to be able to read papers, and later to communicate with colleagues from different countries. As young residents, we were astonished at how seriously she took her English lessons and how rapidly she progressed (Figs. 2 and 3).

And now, another brief departure from a chronological report on Tomilina’s career: when her family returned to Moscow, they moved into an apartment building where almost all the inhabitants were doctors. Perhaps some may not be aware of this, but between 1952 and 1953 many well-known professors of medicine were arrested and imprisoned in Moscow, Leningrad and other big cities, falsely accused of killing their patients. It was Stalin’s famous “doctors’ plot”, resulting in a wave of anti-Semitism, as many of those arrested were Jewish. The families of the doctors arrested lived in terrible isolation because people were afraid to be seen with them. Following the arrest of one of Natalia’s schoolmate’s father (a professor of pathology), Natalia was the only one in her class who continued to visit her friend during those awful months. Fortunately for the doctors (and the country), Stalin died in March 1953. The doctors were released soon afterwards and the accusations against them were dropped.

Tomilina studied General Medicine at Moscow’s second Medical Institute and graduated with distinction in 1961. For a few years she worked as a general practitioner, and then in 1963 moved to the Department of Internal Medicine at the Moscow City Hospital N52, where the first beds for patients with kidney diseases had just been established and started her training in nephrology. Nephrology as a specialization did not exist at the time. It was Natalia’s (and my) mentor, Professor Maria Ratner, who established the first nephrology unit, and it is thanks to her that nephrology developed in Moscow, as well as in the rest of the Soviet Union. Everything was just starting and Tomilina was one of the first professionals in the field.

From 1965 to1968 she worked on her Ph.D. thesis, experimental research on the pathogenesis of edema in nephrotic syndrome in mice [1], defended in 1969 at the Research Institute of Pediatrics. Soon afterwards, a department of nephrology was opened at the Research Institute of Transplantology and Artificial Organs, and in 1969 Tomilina transferred there and started working in clinical transplantology, which again was very new at the time. In 1988 she defended her post-doctoral thesis on transplant nephropathy [2] and became the head of the department mentioned above. In addition to employing new approaches in her work at the hospital, she continued practicing “traditional” nephrology. She was instrumental in introducing kidney biopsy and improving kidney pathology studies. Her interests were very broad indeed: from the physiology and pathophysiology of water and electrolyte balance and tubular dysfunctions to the management of transplant rejection, and from nephropathology to the treatment of idiopathic glomerulonephritis and ANCA-associated vasculitis…. to name a few [3, 4]. In all these years, she never stopped seeing patients with kidney problems and consulting with her residents, interns and Ph.D. students.

Beyond her clinical and scientific ability, Tomilina was an extremely effective healthcare organizer and teacher. In 1993 she organized the Moscow City Nephrology Center, based in City Hospital N52 – probably the first Nephrology Center in the country, and definitely the first one ever with an ICU for kidney patients.

Starting in 1994 and continuing for almost 20 years, she served as chief nephrologist-consultant for the Moscow Healthcare Department. As a chief nephrologist, Tomilina was responsible for the creation of the first peritoneal dialysis program in Moscow (and in the Russian Federation), and played a leading role in organizing peritoneal dialysis services and in education in this field [5]. She also played a crucially important role in the advancement of hemodialysis, and it is thanks to her efforts that access to RRT improved dramatically. Many new HD-units were started, old equipment was replaced in the already existing units, and many ICUs were equipped with dialysis machines. All these steps helped to eliminate the deficit of dialysis places in Moscow, and this approach was gradually expanded to many regions of the Russian Federation.

In 1998, her interest in education on nephrology led Tomilina to found the Russian Dialysis Society, which she headed for 12 years. Under her leadership, RDS organized dozens of educational events throughout Russia and neighboring countries, many of them in collaboration with ERA-EDTA and ISN, thanks to Tomilina’s personal contacts with well-known nephrologists in other countries. She was also the first member of ISN (and probably of ERA-EDTA) to come from the Russian Federation and remained ISN’s Russian representative for almost decade. Another important aspect of the Russian Dialysis Society’s activity is the journal *Nephrology and Dialysis*, an influential Russian-language quarterly, whose editor-in-chief is Tomilina. And finally, she initiated a registry of the patients receiving RRT, which has remained the only source of information about kidney patients in the Russian Federation. The analysis of this database resulted in several publications and participation in DOPPS [6]. In recent years the registry has also provided the ERA-EDTA Registry with data as well.

Tomilina has been a teacher and mentor for several generations of nephrologists. As a teacher, her outstanding quality in my view is her exceptional ability to extract the main point of a case or problem and explain it clearly on the basis of pathophysiology, pathogenesis and clinical course. For many years she taught residents, Ph.D. students, and young nephrologists at point of care, and in 2004 she organized a degree course in nephrology at the Faculty of Postgraduate Education of the Moscow University of Medicine and Dentistry. Chaired by Tomilina, the program of studies includes courses in general nephrology, hemodialysis and peritoneal dialysis, transplantology and nephropathology, which again is a unique combination of courses in postgraduate nephrology education in Russia.

Tomilina is a member of many international societies (ISN, ERA-EDTA, ASN, NKF, ISPD) and the author of numerous papers and contributions to books (a brief list of her publications, representing her wide-ranging scientific interests, is given below). Like other physicians and scientists in the Soviet Union, for many years she was only allowed to publish in Russian journals, which limited international recognition of her research. Starting from the beginning of her professional career, Tomilina has collected and analyzed her clinical experience, and these writings, together with her reflections on selected issues of chronic kidney disease resulted in a book, recently published in Moscow [7].

Nephrology is by no means her only interest. She loves literature and music and has traveled widely, taking thousands of photographs. Since 1952 she has been deeply involved in political life and has a very firm social stand.

To conclude, returning to nephrology, Natalia Tomilina is a clinician, researcher, teacher, organizer, leader, and a real pioneer, who has worked in nephrology from the very beginning of its development in Russia and continues to inspire new generations of Russian nephrologists.
